# Fast and Slow Adaptations of Interlimb Coordination *via* Reflex and Learning During Split-Belt Treadmill Walking of a Quadruped Robot

**DOI:** 10.3389/frobt.2021.697612

**Published:** 2021-08-06

**Authors:** Shinya Aoi, Takashi Amano, Soichiro Fujiki, Kei Senda, Kazuo Tsuchiya

**Affiliations:** ^1^ Department of Aeronautics and Astronautics, Graduate School of Engineering, Kyoto University, Kyoto, Japan; ^2^ Department of Physiology, School of Medicine, Dokkyo Medical University, Tochigi, Japan

**Keywords:** split-belt treadmill walking, quadruped robot, interlimb coordination, spinal reflex, cerebellar learning, central pattern generator

## Abstract

Interlimb coordination plays an important role in adaptive locomotion of humans and animals. This has been investigated using a split-belt treadmill, which imposes different speeds on the two sides of the body. Two types of adaptation have been identified, namely fast and slow adaptations. Fast adaptation induces asymmetric interlimb coordination soon after a change of the treadmill speed condition from same speed for both belts to different speeds. In contrast, slow adaptation slowly reduces the asymmetry after fast adaptation. It has been suggested that these adaptations are primarily achieved by the spinal reflex and cerebellar learning. However, these adaptation mechanisms remain unclear due to the complicated dynamics of locomotion. In our previous work, we developed a locomotion control system for a biped robot based on the spinal reflex and cerebellar learning. We reproduced the fast and slow adaptations observed in humans during split-belt treadmill walking of the biped robot and clarified the adaptation mechanisms from a dynamic viewpoint by focusing on the changes in the relative positions between the center of mass and foot stance induced by reflex and learning. In this study, we modified the control system for application to a quadruped robot. We demonstrate that even though the basic gait pattern of our robot is different from that of general quadrupeds (due to limitations of the robot experiment), fast and slow adaptations that are similar to those of quadrupeds appear during split-belt treadmill walking of the quadruped robot. Furthermore, we clarify these adaptation mechanisms from a dynamic viewpoint, as done in our previous work. These results will increase the understanding of how fast and slow adaptations are generated in quadrupedal locomotion on a split-belt treadmill through body dynamics and sensorimotor integration via the spinal reflex and cerebellar learning and help the development of control strategies for adaptive locomotion of quadruped robots.

## 1 Introduction

Humans and animals change their locomotor behaviors depending on the environment and situation. Interlimb coordination plays an important role in such adaptive locomotion. For example, to walk along a curved path, the outer legs have a longer stride and higher speed than those of the inner legs (Courtine and Schieppati, 2003; Gruntman et al., 2007). Split-belt treadmills, which impose different speeds on the two sides of the body (Yanagihara and Udo, 1994; Prokop et al., 1995; Reisman et al., 2005; Morton and Bastian, 2006; Choi and Bastian, 2007; Frigon et al., 2013), have been used to investigate the mechanisms that control interlimb coordination. Adaptive behaviors induced by changes in the treadmill speed condition have been investigated. During the split-belt treadmill walking of humans, when the treadmill speed condition is changed from the tied configuration (belts move at the same speed) to the split-belt configuration (belts move at different speeds), the relative phase between the leg movements rapidly changes to break the antiphase relationship (i.e., asymmetric interlimb coordination appears) and the stride length and duty factor differ between the two legs (Reisman et al., 2005). However, the relative phase slowly returns to regain the antiphase relationship and to reduce the asymmetric interlimb coordination in the split-belt configuration. The stride length and duty factor remain almost unchanged and different between the two legs. Furthermore, when the treadmill speed condition is returned to the tied configuration, the stride length and duty factor quickly return, whereas the relative phase rapidly diverges from antiphase (i.e., asymmetric interlimb coordination appears even in the tied configuration) and then slowly returns to antiphase to reduce the asymmetry. Because the spinal cord and reflex contribute to rapid changes in locomotor behavior due to environmental changes (Grillner, 1975), it has been suggested that the fast adaptations in split-belt treadmill walking are induced by sensorimotor integration in the spinal cord. The slow changes in the relative phase and the quick divergence of the relative phase from antiphase upon return to the tied configuration do not appear during split-belt treadmill walking of subjects with cerebellar damage (Morton and Bastian, 2006), which suggests that these changes are induced by learning in the cerebellum. In particular, the quick divergence of the relative phase upon return to the tied configuration has been suggested to be the after-effect of learning.

Although these adaptive behaviors are observed in walking on a split-belt treadmill, locomotion is a complicated dynamical phenomenon generated through interactions between the central nervous system, the body’s musculoskeletal system, and the environment, and thus it is difficult to fully understand the locomotion mechanism based on only observations and measurements of the locomotor system. To overcome this limitation, mathematical models and legged robots have been applied to study locomotion (Aoi et al., 2011, 2017; Fukui et al., 2019; Fukuoka et al., 2015; Ijspeert, 2014; Masuda et al., 2021; Otoda et al., 2009; Owaki et al., 2013; Owaki and Ishiguro, 2017; Spröwitz et al., 2013). In our previous works (Fujiki et al., 2013, 2015), we developed a locomotion control system for a biped robot based on the spinal reflex and cerebellar learning. We reproduced the fast and slow adaptive behaviors observed in humans during split-belt treadmill walking of the robot. These behaviors were not the result of specifically designed features in our control system, but emerged through the body dynamics and sensorimotor integration via the spinal reflex and cerebellar learning. We clarified these adaptation mechanisms from a dynamic viewpoint by focusing on the changes in the relative positions between the center of mass and foot stance induced by reflex and learning.

Quadrupeds such as cats and mice also exhibit fast and slow changes in interlimb coordination during split-belt treadmill walking (D’Angelo et al., 2014; Darmohray et al., 2019; Frigon et al., 2013; Yanagihara and Udo, 1994). Interlimb coordination in quadrupedal locomotion is more complicated than that in human locomotion due to the increased number of legs. Rapid changes have been observed in spinal cats (Forssberg et al., 1980; Frigon et al., 2013) and slow changes and the after-effect do not appear in mice with cerebellar dysfunction (Darmohray et al., 2019), which suggest that the spinal reflex and cerebellar learning contribute to fast and slow adaptations, respectively, during split-belt treadmill walking of quadrupeds, as is the case for humans. Although previous works (Ito et al., 1998; Kodono and Kimura, 2020; Latash et al., 2020) have investigated adaptive quadrupedal locomotion on a split-belt treadmill using mathematical models and legged robots, they considered specific conditions [e.g., only one of the four legs moved at a different speed (Ito et al., 1998; Kodono and Kimura, 2020) and only the center of mass dynamics in the frontal plane were considered (Latash et al., 2020)]. The gait adaptation mechanism in quadrupedal locomotion through whole-body dynamics and sensorimotor integration for different left- and right-side speeds remains unclear.

In this study, we improve our locomotion control system for a biped robot and apply it to a quadruped robot. We demonstrate that although the basic gait pattern of our robot is different from that of general quadrupeds (due to limitations of the robot experiment), fast and slow adaptations similar to those of quadrupeds appear during split-belt treadmill walking of the quadruped robot. Furthermore, we clarify the adaptation mechanisms from a dynamic viewpoint, as done in our previous work (Fujiki et al., 2015). These results will increase the understanding of how fast and slow adaptations are generated in quadrupedal locomotion on a split-belt treadmill through body dynamics and sensorimotor integration via the spinal reflex and cerebellar learning and help the development of control strategies for adaptive locomotion of quadruped robots.

## 2 Materials and Methods

### 2.1 Quadruped Robot

**FIGURE 1:**
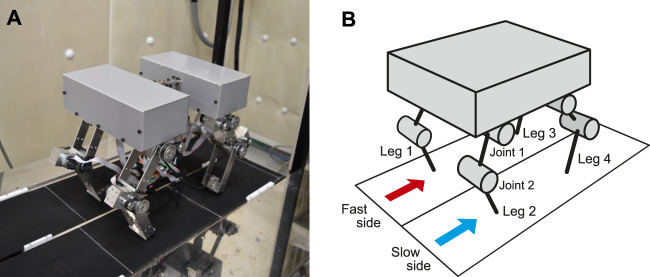
Experimental setup. **(A)** Photograph of quadrupedal robot on split-belt treadmill. The robot body consists of two sections that are mechanically attached to each other. **(B)** Schematic model of quadrupedal robot.

In this study, we used the quadruped robot (Figure 1) developed in our previous work (Aoi et al., 2013a). It consists of a body and four legs (Legs 1–4). Each leg consists of two links connected by pitch joints (Joints 1 and 2), with each joint manipulated by a motor. A touch sensor is attached to the tip of each leg.

Electric power is externally supplied and the robot is controlled by an external host computer (Intel Pentium 4 2.8 GHz, RT-Linux), which calculates the desired joint motions and solves the oscillator phase dynamics in the control system (see Section 2.3). The robot receives command signals at intervals of 1 ms. It is connected to the electric power unit and the host computer by cables, which are slack and suspended during the experiment to avoid influencing the robot’s locomotor behavior.

### 2.2 Split-Belt Treadmill

The robot walked on the split-belt treadmill (Figure 1) developed in our previous works (Fujiki et al., 2013, 2015). The treadmill has two parallel belts, each of which is equipped with a motor and an encoder to control the individual belt speed. The width of each belt is 15 cm and the distance between rotation axes is 64 cm.

### 2.3 Locomotion Control System

**FIGURE 2:**
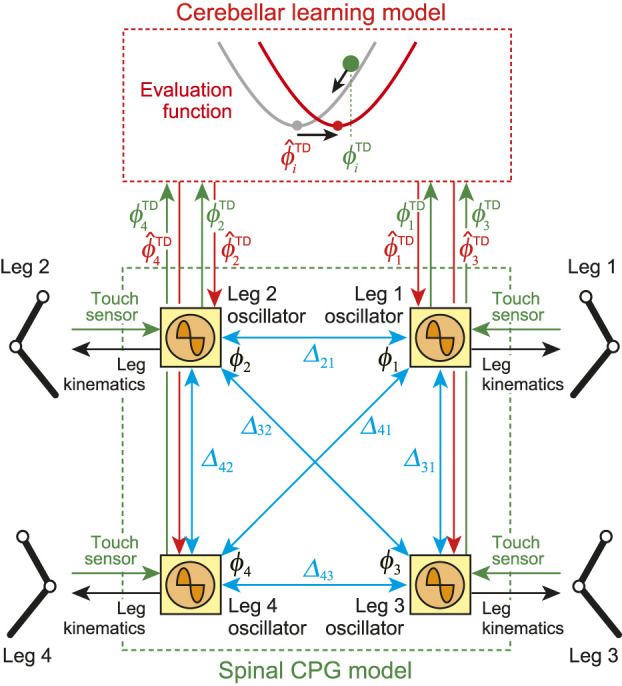
Locomotion control model composed of spinal CPG and cerebellar learning models. Spinal model consists of four phase oscillators (Leg 1–4 oscillators). Blue arrows indicate relative phase ${\text{Δ}}_{ij}$ between oscillators. Oscillator phases are modulated by phase resetting based on touch sensor signals (green arrows) and desired (predicted) touchdown timing (red arrows). Oscillator phases determine leg kinematics (black arrows). Cerebellar model receives touchdown phase (green arrows) and modifies desired (predicted) touchdown phase using evaluation function, which is sent to spinal model (red arrows).

In our previous work (Fujiki et al., 2015), we developed a locomotion control system for a biped robot based on the spinal and cerebellum functions. In this study, we improved the control system and applied it to the quadruped robot (Figure 2). The control system consists of spinal and cerebellum models. The spinal model produces motor commands to manipulate the robot based on a central pattern generator (CPG) and the sensory reflex, and the cerebellum model modulates motor commands through learning.

#### 2.3.1 Spinal CPG Model

Our spinal CPG model is based on a physiological two-layer network model composed of rhythm generator (RG) and pattern formation (PF) networks (Burke et al., 2001; Rybak et al., 2006). The RG network creates the basic rhythm. It alters the rhythm by producing phase shifts and by performing rhythm resetting in response to sensory feedback (phase resetting). The PF network shapes the rhythm into spatiotemporal motor command patterns. Based on this physiological finding, we developed the spinal CPG model using the following RG and PF models.

For the RG model, we used four simple phase oscillators (Leg 1–4 oscillators), whose phases are denoted by $\phi_{i}$ (i=1,…,4, $0\leq \phi_{i}<2\pi$). Because the oscillator phase determines the desired movement of the corresponding leg, as explained below, the relative phases between the oscillators ${\text{Δ}}_{ij}=\phi_{i}-\phi_{j}$ (i,j=1,…,4, $0\leq {\text{Δ}}_{ij}<2\pi$) determine the gait. The oscillator phases follow the dynamics

$${\dot{\phi }}_{i}=\omega -\sum_{j=1}^{4}K_{ij}\text{sin}\left({\text{Δ}}_{ij}-{\hat{\text{Δ}}}_{ij}\right)+\left({\hat{\phi }}_{i}^{\text{TD}}-\phi_{i}^{\text{TD}}\right)\delta \left(t-t_{i}^{\text{TD}}\right)$$ (1)

where *ω* is the basic oscillator frequency, $K_{ij}$ is the gain parameter, and δ(⋅) is the Dirac delta function. The second term on the right-hand side represents the interactions among oscillators to move the relative phase ${\text{Δ}}_{ij}$ to the desired value ${\hat{\text{Δ}}}_{ij}$, which is determined by the desired gait pattern. The third term on the right-hand side represents phase resetting. Taking inspiration from spinal cats walking on a treadmill, which show how touchdown information influences the locomotion phase and rhythm generated by a CPG (Duysens et al., 2000), we modulate the oscillator phase so that it responds to touch sensor signals based on phase resetting. Specifically, when the touchdown of Leg *i* occurs at time $t_{i}^{\text{TD}}$ ($\phi_{i}=\phi_{i}^{\text{TD}}$ at $t_{i}^{\text{TD}}$), the phase of Leg *i* oscillator $\phi_{i}$ is reset from $\phi_{i}^{\text{TD}}$ to ${\hat{\phi }}_{i}^{\text{TD}}$ (superscript TD refers to touchdown). This ${\hat{\phi }}_{i}^{\text{TD}}$ corresponds to the desired (predicted) touchdown phase, as explained in Section 2.3.2.

**FIGURE 3:**
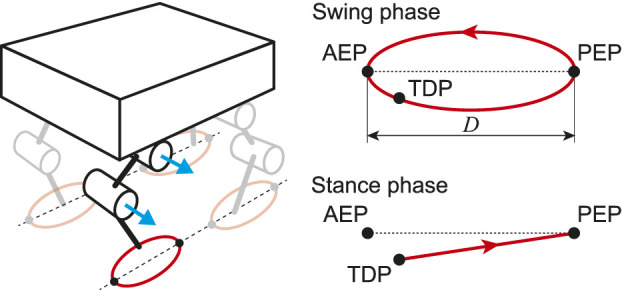
Desired leg kinematics composed of swing and stance phases. At touchdown position (TDP), trajectory changes from swing to stance phase. When leg tip reaches posterior extreme position (PEP), trajectory moves into swing phase. Anterior extreme position (AEP) is desired position of touchdown.

For the PF model, taking inspiration from the physiological finding that spinocerebellar neurons encode the global information of limb kinematics, such as the length and orientation of the limb axis (Bosco and Poppele, 2001; Poppele et al., 2002; Poppele and Bosco, 2003), we produced the motor commands to achieve the desired leg kinematics of the robot based on the oscillator phases obtained from the RG model. We use simple leg kinematics, which consist of the swing and stance phases (Figure 3), in reference to the length and orientation of the limb axis in the pitch plane. The swing phase uses a simple closed curve for the leg tip that includes the anterior extreme position (AEP) and the posterior extreme position (PEP). It starts from the PEP and continues until touchdown. The AEP corresponds to the desired position of touchdown. The stance phase uses a straight line from the touchdown position (TDP) to the PEP. The trajectories for the swing and stance phases are given as functions of the corresponding oscillator phase, where $\phi_{i}=0$ at the PEP and $\phi_{i}={\hat{\phi }}_{i}^{\text{TD}}$ at the AEP. We denote the distance between the AEP and PEP as *D* and the gait cycle as *T* (ω=2π/T). The desired duty factor ${\hat{\beta }}_{i}$, stride length ${\hat{S}}_{i}$, and locomotion speed ${\hat{V}}_{i}$ of Leg *i* are then given by

$${\hat{\beta }}_{i}=1-\frac{{\hat{\phi }}_{i}^{\text{TD}}}{2\pi },{\hat{S}}_{i}=\frac{D}{{\hat{\beta }}_{i}},{\hat{V}}_{i}=\frac{D}{{\hat{\beta }}_{i}T}$$ (2)

To generate the desired kinematics, the desired joint trajectories are calculated based on the inverse kinematics and each joint is controlled by the joint torque based on proportional-derivative feedback control.

#### 2.3.2 Cerebellar Learning Model

The cerebellum predicts the sensory consequences of movement based on the efference copy, and modifies motor commands to reduce errors between the predicted and actual sensory information through learning. Furthermore, it predicts the timing of sensory events (Nixon and Passingham 2001; O’Reilly et al. 2008) and contributes to achieving tasks that require accurate temporal control (Ivry et al., 2012; Ivry and Keele, 1989; Spencer et al., 2005). During walking on a surface with an unexpected hole, the absence of touchdown sensory feedback at the predicted timing triggers reflex-like reaction behavior (Hiebert et al., 1994; van der Linden et al., 2007), which suggests that the prediction of touchdown timing is important for motor learning in walking.

We focused on touchdown timing for the cerebellar model. In particular, we modulate the desired (predicted) touchdown timing ${\hat{\phi }}_{i}^{\text{TD}}$ based on the error between the predicted and actual touchdown timings. For this purpose, we define an evaluation function $E_{i,n}$ for the *n*th step of Leg *i* using the error between the predicted touchdown phase ${\hat{\phi }}_{i,n}^{\text{TD}}$ and the actual touchdown phase $\phi_{i,n}^{\text{TD}}$ for the *n*th step of Leg *i*, which is given by

$$E_{i,n}=\frac{1}{2}{\left({\hat{\phi }}_{i,n}^{\text{TD}}-\phi_{i,n}^{\text{TD}}\right)}^{2}$$ (3)

Based on this evaluation function, we predict the next touchdown timing. Specifically, from the gradient direction of the evaluation function, ${\hat{\phi }}_{i}^{\text{TD}}$ is modulated by

$${\hat{\phi }}_{i,n+1}^{\text{TD}}={\hat{\phi }}_{i,n}^{\text{TD}}-\alpha \frac{\partial E_{i,n}}{\partial {\hat{\phi }}_{i,n}^{\text{TD}}}$$ (4)

where *α* is the learning rate. Because ${\hat{\phi }}_{i}^{\text{TD}}$ is the desired timing of the corresponding leg to switch from the swing phase to the stance phase, this temporal modulation changes the desired duty factor of the corresponding leg (Eq. 2). Therefore, if the touchdown arrives earlier than predicted, the robot increases the swing leg speed in the next step (${\hat{\phi }}_{i}^{\text{TD}}$ decreases and ${\hat{\beta }}_{i}$ increases while *D* remains unchanged). In addition, the TDP gravitates to alignment with the AEP (Figure 3) through this modulation.

### 2.4 Robot Experiment

To clarify the functional roles of the spinal and cerebellar models in gait adaptation during split-belt treadmill walking of the quadruped robot, we considered the following two cases in the robot experiment: 1) with the spinal model but without the cerebellar model, that is, the desired (predicted) touchdown timing ${\hat{\phi }}_{i}^{\text{TD}}$ (i=1,…,4) was fixed, and 2) with both the spinal and cerebellar models. For both cases, we suddenly changed the treadmill speed condition during walking and investigated how the locomotor behavior changed. In particular, when we used only the spinal model, we investigated fast adaptation via the sensory reflex using various treadmill speed conditions. In contrast, when we used both the spinal and cerebellar models, we examined slow adaptation via learning as well as fast adaptation.

For the quadruped robot, we used the following control parameters: D=1.5 cm, T=0.33 s, and α=0.25. For the initial value of ${\hat{\phi }}_{i}^{\text{TD}}$, we used *π*, which gives ${\hat{\beta }}_{i}=0.5$, ${\hat{S}}_{i}=3$ cm, and ${\hat{V}}_{i}=9.1$ cm/s. For the desired value of the relative phases, we used ${\hat{\text{Δ}}}_{12}={\hat{\text{Δ}}}_{34}=\pi$ and ${\hat{\text{Δ}}}_{13}={\hat{\text{Δ}}}_{24}=0$. This means that the desired gait pattern was the pace pattern, which is different from the walk pattern (${\hat{\text{Δ}}}_{13}={\hat{\text{Δ}}}_{24}=-\pi /2$) and trot pattern (${\hat{\text{Δ}}}_{13}={\hat{\text{Δ}}}_{24}=\pi$) of general quadrupeds. This was done because the robot with the walk or trot pattern could not continue walking straight on the split-belt treadmill. Specifically, it easily changed walking direction (yaw motion was induced) due to changes in the treadmill speed condition because the fore and hind legs were in contact with different belts. Instead, we used small values for $K_{ij}$ ($K_{12}=K_{21}=K_{34}=K_{43}=K_{13}=K_{31}=K_{24}=K_{42}=2$, with other $K_{ij}$ set to zero) so that the relative phases could be shifted from the desired value by phase resetting and learning through locomotion dynamics [we used 20 for $K_{ij}$ to fix the relative phase to the desired value in our previous work (Aoi et al., 2013a)]. The same control parameters and initial conditions were used irrespective of the use of the cerebellar model and treadmill speed condition.

For the split-belt treadmill, we used the tied configuration at the beginning of the robot walk with $v_{1}=v_{2}=6.5$ cm/s, where $v_{1}$ and $v_{2}$ are the speeds of the right belt (Legs 1and 3) and left belt (Legs 2 and 4), respectively. After the robot had established a steady gait, we suddenly changed the speed condition from the tied configuration to the split-belt configuration, but did not change the control strategy and parameters. When we used only the spinal model, we used the following three speed conditions for the split-belt configuration: 3x: $v_{1}=9.8$ and $v_{2}=3.3$ cm/s ($v_{1}/v_{2}=3$), 4x: $v_{1}=10.8$ and $v_{2}=2.7$ cm/s ($v_{1}/v_{2}=4$), 5x: $v_{1}=13.5$ and $v_{2}=2.7$ cm/s ($v_{1}/v_{2}=5$). Therefore, we consider Legs 1 and 3 as the fast side and Legs 2 and 4 as the slow side (Figure 1B). When we incorporated the cerebellar model, we used the 5x condition for the split-belt configuration. In addition, we returned the speed condition to the tied configuration from the split-belt configuration without changing the control strategy and parameters.

We performed these robot trials five times for each speed condition and investigated the robot’s behavior from the averages of the results for six steps in each configuration period. When we incorporated the cerebellar model, we separated the periods of the split-belt configuration and the second tied configuration into two halves to clarify early and late stages of adaptation in each period. We used one-way repeated-measures analysis of variance (ANOVA) to compare the differences between the periods and to clarify the significance of the locomotor behavior changes. When the ANOVA results showed a significant difference, we conducted post hoc analysis using Tukey’s honestly significant difference test, where we considered that p<0.05 indicates a significant difference.

## 3 Results

### 3.1 Fast Adaptation by Reflex

We first used only the spinal model for the robot experiment. At the beginning, the robot walked on the treadmill in the tied configuration with the fore and hind legs in contact with the ipsilateral belt. It continued walking after the treadmill speed condition changed to the 3x, 4x, and 5x conditions of the split-belt configuration. Note that when we did not use phase resetting in Eq. 1, the robot could not walk on the treadmill even in the tied configuration.

**FIGURE 4:**
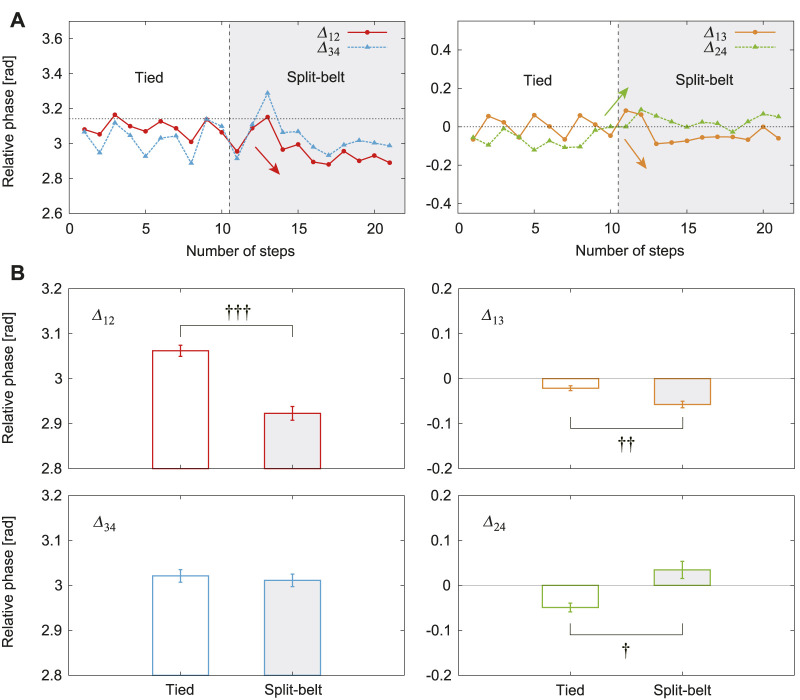
Relative phases between leg oscillators with use of only spinal model. **(A)** ${\text{Δ}}_{12}$, ${\text{Δ}}_{13}$, ${\text{Δ}}_{24}$, and ${\text{Δ}}_{34}$ for one representative trial of 5x condition. **(B)** Their averages for tied and split-belt configurations. Data points and error bars are the mean and standard error results of five experiments, respectively. †:p<0.05, ††:p<0.01, and †††:p<0.001.

Figure 4A shows the relative phases ${\text{Δ}}_{12}$, ${\text{Δ}}_{13}$, ${\text{Δ}}_{24}$, and ${\text{Δ}}_{34}$ for one representative trial of the 5x condition using the average value for one gait cycle obtained using $\frac{1}{T}\int_{T}{\text{Δ}}_{ij}dt$ (see supplementary movie). ${\text{Δ}}_{12}$ and ${\text{Δ}}_{34}$ were almost *π* and ${\text{Δ}}_{13}$ and ${\text{Δ}}_{24}$ were almost 0 in the tied configuration. ${\text{Δ}}_{12}$ and ${\text{Δ}}_{13}$ decreased and ${\text{Δ}}_{24}$ increased in the split-belt configuration. Figure 4B shows their averages in the tied configuration and the split-belt configuration in the 5x condition for five trials, where we used six steps for each configuration period in one trial. ${\text{Δ}}_{13}$ and ${\text{Δ}}_{24}$ showed significant differences between the belt speed conditions (p<0.01 and p<0.05, respectively) and ${\text{Δ}}_{12}$ showed the most significant difference (p<0.001). In contrast, ${\text{Δ}}_{34}$ showed no significant difference.

**FIGURE 5:**
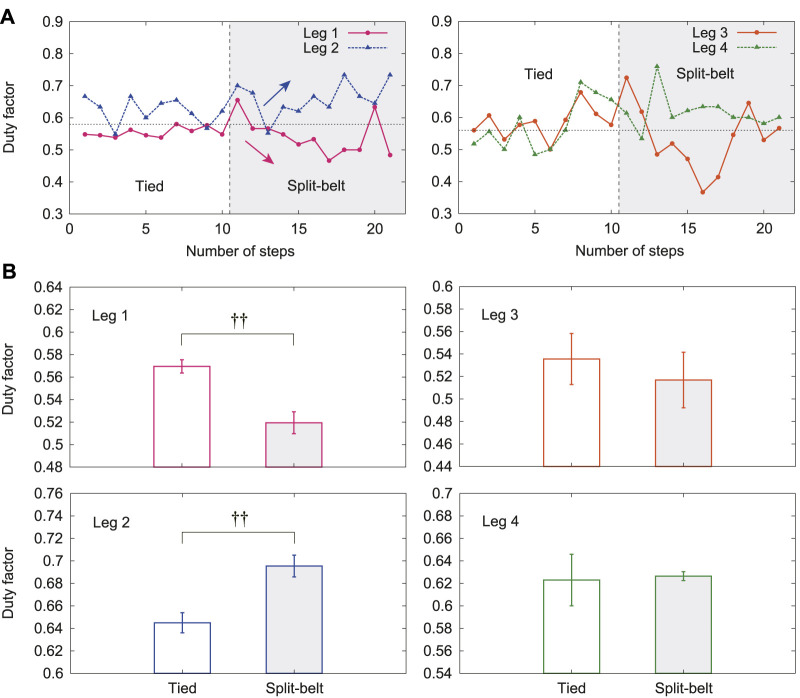
Duty factors with use of only spinal model. **(A)** Duty factors for Legs 1–4 for one representative trial of 5x condition. **(B)** Their averages for tied and split-belt configurations. Data points and error bars are the mean and standard error results of five experiments, respectively. ††:p<0.01.

Figure 5A shows the duty factors of Legs 1–4 for one representative trial of the 5x condition. The duty factors of Legs 1 and 2 were around 0.6 in the tied configuration. That of Leg 1 decreased and that of Leg 2 increased in the split-belt configuration. In contrast, those of Legs 3 and 4 slightly fluctuated around 0.6 and did not show clear trends. Figure 5B shows their averages in the tied configuration and the split-belt configuration in the 5x condition for five trials. The duty factors of Legs 1 and 2 showed significant differences between the belt speed conditions (both p<0.01), whereas those of Legs 3 and 4 showed no significant difference.

**FIGURE 6:**
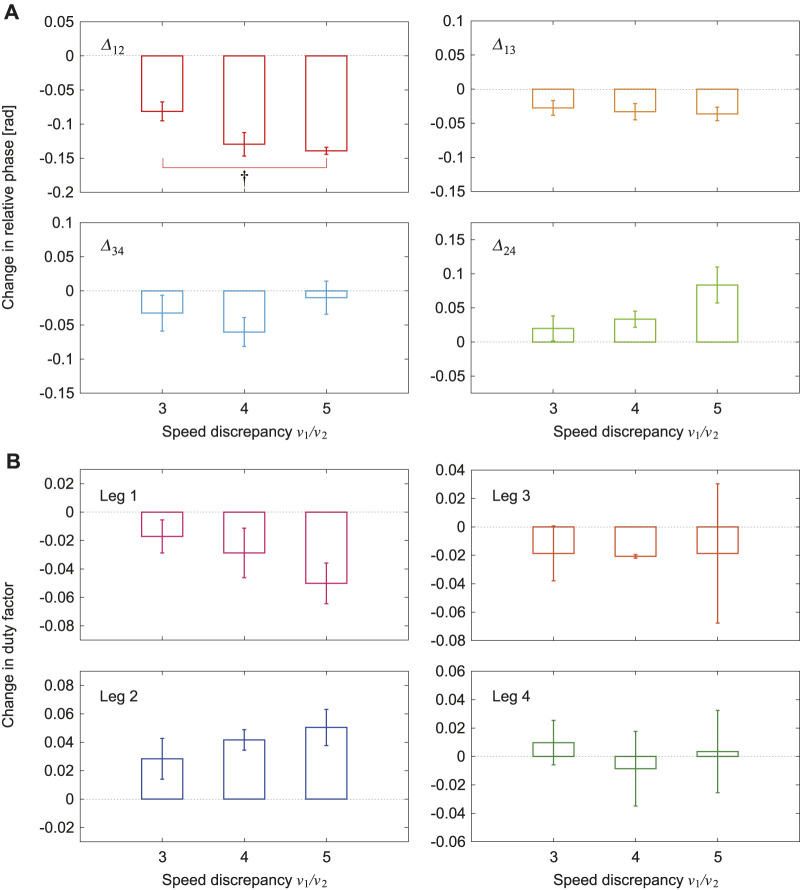
Changes in average **(A)** relative phases and **(B)** duty factors between tied and split-belt configurations for 3x, 4x, and 5x conditions ($v_{1}/v_{2}=3$, 4, and 5). Data points and error bars are the mean and standard error results of five experiments, respectively. †:p<0.05.

Figures 6A, B show the changes in the average relative phases and duty factors, respectively, between the tied configuration and the split-belt configuration for three speed conditions (3x, 4x, and 5x). The changes in the relative phases ${\text{Δ}}_{13}$ and ${\text{Δ}}_{34}$ showed no clear dependence on the speed condition, whereas those in ${\text{Δ}}_{12}$ and ${\text{Δ}}_{24}$ increased as the speed discrepancy between the left and right belts increased. In particular, the change in ${\text{Δ}}_{12}$ showed a significant difference between the 3x and 5x conditions (p<0.05). The changes in the duty f\actors for Legs 3 and 4 showed no clear dependence on the speed condition, whereas those for Legs 1 and 2 increased as the speed discrepancy between the left and right belts increased. However, they showed no significant difference.

### 3.2 Slow Adaptation by Learning

We next used both the spinal and cerebellar models for the robot experiment. At the beginning, the robot walked on the treadmill in the tied configuration. It continued walking when the treadmill speed condition changed to the 5x condition of the split-belt configuration. Furthermore, the robot continued walking after the treadmill speed condition returned to the tied configuration.

**FIGURE 7:**
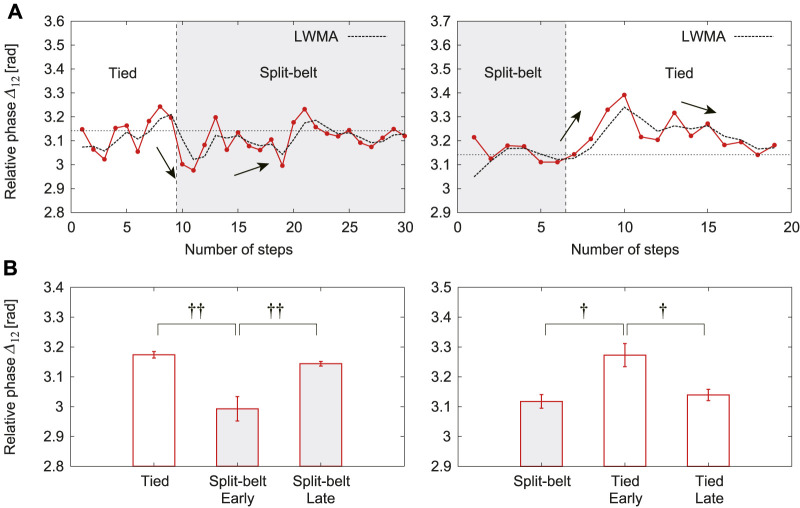
Relative phase ${\text{Δ}}_{12}$ with use of both spinal and cerebellar models. **(A)** One representative trial with moving average [five-period linear weighted moving average (LWMA)]. **(B)** Average for each period. Data points and error bars are the mean and standard error results of five experiments, respectively. †:p<0.05 and ††:p<0.01.

The relative phase ${\text{Δ}}_{12}$ clearly changed depending on the treadmill speed condition, similar to previous results, whereas the other relative phases did not. Figure 7A shows ${\text{Δ}}_{12}$ for one representative trial, where the left and right figures show the results from the first tied configuration to the split-belt configuration and from the split-belt configuration to the second tied configuration, respectively. ${\text{Δ}}_{12}$ was almost *π* in the first tied configuration. It quickly decreased at the early stage (first half) of the split-belt configuration and slowly returned to *π* at the late stage (last half). In addition, it quickly decreased at the early stage of the second tied configuration, which suggests the after-effect of learning. Finally, it slowly returned to *π* at the late stage of the second tied configuration. Figure 7B shows the average in the first tied configuration and early and late stages of the split-belt and second tied configurations. Significant differences appear between the first tied configuration and early stage of the split-belt configuration (p<0.01), between the early and late stages of the split-belt configuration (p<0.01), between the split-belt configuration and the early stage of the second tied configuration (p<0.05), and between the early and late stages of the second tied configuration (p<0.05).

**FIGURE 8:**
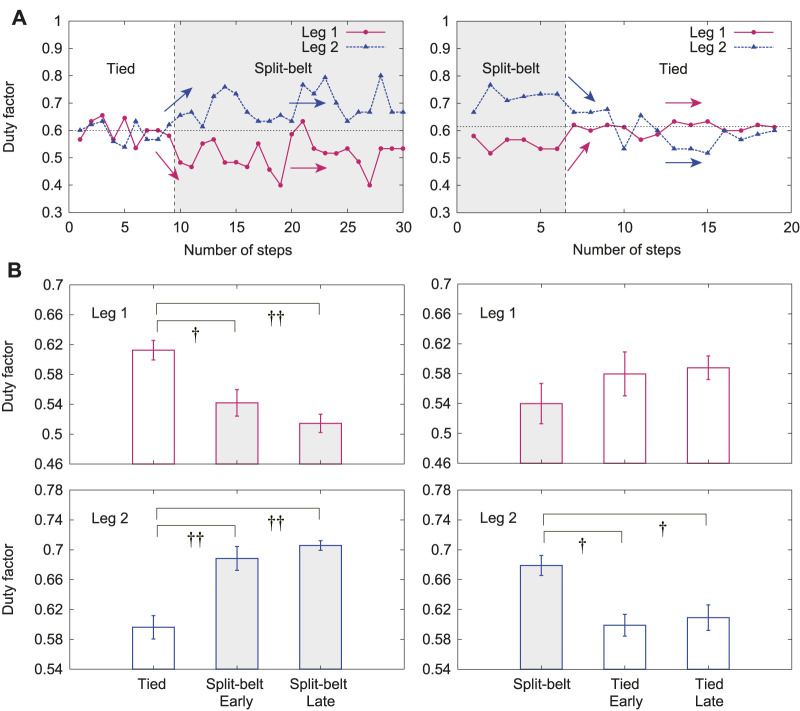
Duty factors for Legs 1 and 2 with use of both spinal and cerebellar models. **(A)** One representative trial. **(B)** Averages for each period. Data points and error bars are the mean and standard error results of five experiments, respectively. †:p<0.05 and ††:p<0.01.

In this experiment, the duty factors for Legs 1 and 2 clearly changed depending on the treadmill speed condition, whereas the other duty factors did not, similar to previous results. Figure 8A shows the duty factors for Legs 1 and 2 for one representative trial, where the left and right figures show the results from the first tied configuration to the split-belt configuration and from the split-belt configuration to the second tied configuration, respectively. The duty factors for Legs 1 and 2 were almost 0.6 in the first tied configuration. The duty factor for Leg 1 quickly decreased and that for Leg 2 increased at the early stage of the split-belt configuration. However, they had almost no change at the late stage, unlike the relative phases (Figure 7). They quickly returned to almost 0.6 at the early stage of the second tied configuration and did not change at the late stage. Figure 8B shows their averages for the first tied configuration and the early and late stages for the split-belt and second tied configurations. The duty factor for Leg 1 showed significant differences between the first tied configuration and early stage of the split-belt configuration (p<0.05) and between the first tied configuration and late stage of the split-belt configuration (p<0.01). However, it showed no significant difference between the split-belt configuration and early and late stages of the second tied configuration. The duty factor for Leg 2 also showed significant differences between the first tied configuration and early stage of the split-belt configuration (p<0.01) and between the first tied configuration and late stage of the split-belt configuration (p<0.01). In addition, it showed significant differences between the split-belt configuration and early stage of the second tied configuration (p<0.05) and between the split-belt configuration and early stage of the second tied configuration (p<0.05).

## 4 Discussion

### 4.1 Fast Adaptation Mechanism Upon Change to Split-Belt Configuration

When we used only the spinal model, the relative phase ${\text{Δ}}_{34}$ exhibited almost no change, whereas ${\text{Δ}}_{12}$ decreased from *π*, ${\text{Δ}}_{13}$ decreased from 0, and ${\text{Δ}}_{24}$ increased from 0 due to the change in the treadmill speed condition from the tied configuration to the split-belt configuration (Figure 4). The duty factor for Leg 1 increased, that for Leg 2 decreased, and those for Legs 3 and 4 exhibited almost no change (Figure 5). The asymmetric interlimb coordination and duty factors allow the robot to walk in the asymmetric speed condition. Furthermore, these asymmetries increased as the belt speed discrepancy increased (Figure 6). Such asymmetric locomotion parameters and increase in asymmetries induced by the speed condition have been observed in cats and mice (D’Angelo et al., 2014; Darmohray et al., 2019). Our results are consistent with these observations. Note that the fast changes in our robot were not the result of specifically designed features in our control system, but emerged through the body dynamics and sensorimotor integration via the spinal reflex. We discuss the mechanism of these gait adaptations from a dynamic viewpoint by focusing on changes in the foot contact timing because the locomotor behavior is modulated by phase resetting in Eq. 1 based on foot contact timing in the spinal model, where we assume that the forward/backward movements and pitch rotation are dominant, as assumed in our previous work on a biped robot (Fujiki et al., 2015), because the backward speed of the treadmill belts changes.

**FIGURE 9:**
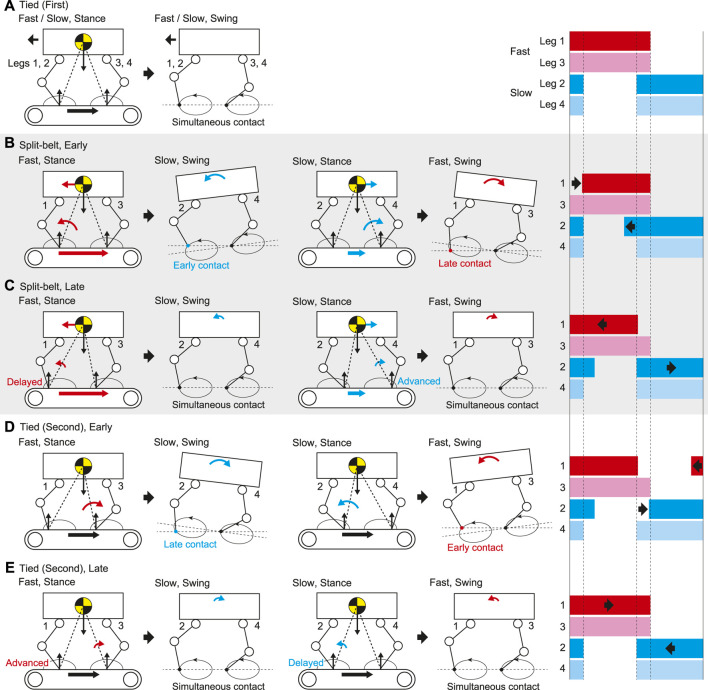
Gait adaptation mechanism through reflex and learning based on foot contact timing in **(A)** first tied, **(B)** early stage of split-belt, **(C)** late stage of split-belt, **(D)** early stage of second tied, and **(E)** late stage of second tied configurations. Right figures show foot diagrams.

In the tied configuration, the stance legs on both the fast and slow sides are pulled at the same speed. The fore and hind legs on the ipsilateral side contact the belt simultaneously (Figure 9A). In contrast, the stance legs on the fast side (Legs 1 and 3) are strongly pulled in the split-belt configuration, which accelerates the body and tilts it forward (Figure 9B). As a result, the fore leg on the slow side (Leg 2) touches the belt earlier than in the tied configuration. However, the foot contact timing of the hind leg on the slow side (Leg 4) shows almost no change because the swing leg trajectory moves upward due to the body tilt while the anterior part of the trajectory moves downward due the trajectory tilt. The stance legs on the slow side (Legs 2 and 4) are weakly pulled in the split-belt configuration, which decelerates the body and tilts it backward. As a result, the fore leg on the fast side (Leg 1) touches the belt later than in the tied configuration. However, the foot contact timing of the hind leg on the fast side (Leg 3) shows almost no change because the swing leg trajectory moves downward due to the body tilt while the anterior part of the trajectory moves upward due the trajectory tilt. These changes in the foot contact timings change the relative phases ${\text{Δ}}_{12}$, ${\text{Δ}}_{13}$, and ${\text{Δ}}_{24}$, and the duty factors for Legs 1 and 2, without changing the relative phase ${\text{Δ}}_{34}$ and the duty factors for Legs 3 and 4. As the speed discrepancy between the belts increases, changes in the body tilt and foot contact timings increase. As a result, the changes in the relative phases and duty factors increase.

### 4.2 Slow Adaptation Mechanism in Split-Belt Configuration

When we incorporated the cerebellar model as well as the spinal model, adaptive changes in locomotor behaviors similar to those observed with the use of only the spinal model appeared at the early stage of the split-belt configuration. However, different adaptive behavior appeared in the late stage of the split-belt configuration due to the learning by the cerebellar model. In particular, after the relative phase ${\text{Δ}}_{12}$ quickly decreased from *π* at the early stage, it slowly returned to *π* at the late stage; that is, the asymmetry in the interlimb coordination was slowly reduced (Figure 7). In contrast, although the duty factors for Legs 1 and 2 quickly decreased and increased, respectively, at the early stage, they remained almost unchanged at the late stage (Figure 8). Such a slow reduction of the asymmetry in interlimb coordination has been observed in cats and mice (Darmohray et al., 2019; Yanagihara and Udo, 1994). Our results are consistent with these observations. Note that the changes in the locomotor behavior at the late stage for our robot were not characteristics that we specifically designed into our control model, but were generated through the body dynamics and sensorimotor integration via the spinal reflex and cerebellar learning. We discuss the mechanism of these gait adaptations from a dynamic viewpoint, as done in the previous section, by focusing on changes in foot contact timings because the locomotor behavior is also modulated by the cerebellar learning model Eq. 4 based on foot contact timing through phase resetting in Eq. 1.

Because the touchdown of the fore leg on the slow side (Leg 2) is advanced at the early stage (Figure 9B), the swing leg speed slowly increases due to learning. As a result, the stance legs on the fast side (Legs 1 and 3) are delayed relative to the slow side at the late stage, which reduces the pitching moment to tilt the body forward and induces simultaneous foot contact between the fore and hind legs on the slow side (Legs 2 and 4), as shown in Figure 9C. Similarly, because the touchdown of the fore leg on the fast side (Leg 1) is delayed at the early stage (Figure 9B), the swing leg speed slowly decreases due to learning. As a result, the stance legs on the slow side (Legs 2 and 4) are advanced relative to the fast side at the late stage, which reduces the pitching moment to tilt the body backward and induces simultaneous foot contact between the fore and hind legs on the fast side (Legs one and 3), as shown in Figure 9C. These slow changes in the foot contact timings change the relative phase ${\text{Δ}}_{12}$ without changing the duty factors at the late stage. Note that although this mechanism suggests that ${\text{Δ}}_{13}$ and ${\text{Δ}}_{24}$ also show further changes at the late stage, we did not clearly observe such changes because they are smaller than those for ${\text{Δ}}_{12}$, as shown in Figure 9C.

### 4.3 After-Effect Mechanism due to Fast and Slow Adaptations Upon Return to Tied Configuration

When the treadmill speed condition was returned to the tied configuration, locomotor behaviors different from those in the first tied configuration appeared. In particular, the relative phase ${\text{Δ}}_{12}$ quickly diverged from *π*; that is, the asymmetry in interlimb coordination appeared again (Figure 7). Although this quick change is due to the spinal reflex, the divergence from *π* is due to learning in the previous split-belt configuration. This suggests the after-effect of learning. In contrast, the duty factors for Legs 1 and 2 returned to the values in the first tied configuration (Figure 8). Such asymmetry in the interlimb coordination due to the after-effect has been observed in cats and mice (Darmohray et al., 2019; Yanagihara and Udo, 1994). Our results are consistent with these observations. Note that these changes in our robot were not the result of specifically designed features in our control system, but emerged through the body dynamics and sensorimotor integration via the spinal reflex and cerebellar learning. We discuss the mechanism of these gait adaptations from a dynamic viewpoint, as done in previous sections.

In the late stage of the split-belt configuration, the stance legs on the fast side (Legs 1 and 3) are delayed relative to the slow side due to the learning effect, which reduces the pitching moment to tilt the body forward by the strong pulling (Figure 9C). When the treadmill speed condition is returned to the tied configuration, the strong pulling also returns, which induces the pitching moment to tilt the body backward (Figure 9D). As a result, the fore leg on the slow side (Leg 2) touches the belt later than in the late stage of the split-belt configuration. The foot contact timing of the hind leg on the slow side (Leg 4) shows almost no change for the same reason as that for the foot contact timing of the hind leg on the fast side (Leg 3) in the early stage of the split-belt configuration (Figure 9B). Similarly, in the late stage of the split-belt configuration, the stance legs of the slow side (Legs 2 and 4) are advanced relative to the fast side due to the learning effect, which reduces the pitching moment to tilt the body backward by the weak pulling (Figure 9C). When the treadmill speed condition is returned to the tied configuration, the weak pulling also returns, which induces the pitching moment to tilt the body forward (Figure 9D). As a result, the fore leg on the fast side (Leg 1) touches the belt earlier than in the late stage of the split-belt configuration. The foot contact timing of the hind leg on the fast side (Leg 3) shows almost no change for the same reason as that for the foot contact timing of the hind leg on the slow side (Leg 4) in the early stage of the split-belt configuration (Figure 9B). These changes in the foot contact timings induce a different behavior of ${\text{Δ}}_{12}$ from that in the first tied configuration and the same behaviors of the duty factors for Legs 1 and 2 as those in the first tied configuration.

### 4.4 Slow Adaptation Mechanism After Return to Tied Configuration

Although the relative phase ${\text{Δ}}_{12}$ showed behavior at the early stage of the second tied configuration different from that in the first tied configuration due to the after-effect, it slowly returned at the late stage through learning (Figure 7). That is, the asymmetry in interlimb coordination appeared at the early stage and slowly reduced at the late stage. The slow reduction of the asymmetry in interlimb coordination induced by the after-effect has been observed in cats and mice (Darmohray et al., 2019; Yanagihara and Udo, 1994). Our results are consistent with these observations. Note that these changes in our robot were not characteristics that we specifically designed into our control model, but were generated through the body dynamics and sensorimotor integration via the spinal reflex and cerebellar learning. We discuss the mechanism of this gait adaptation from a dynamic viewpoint, as done in previous sections.

Because the touchdown of the fore leg on the slow side (Leg 2) is delayed at the early stage (Figure 9D), the swing leg speed slowly decreases due to learning. As a result, the stance legs on the fast side (Legs 1 and 3) are advanced relative to the slow side at the late stage, which reduces the pitching moment to tilt the body backward and induces simultaneous foot contact between the fore and hind legs on the slow side (Legs 2 and 4), as shown in Figure 9E. Similarly, because the touchdown of the fore leg on the fast side (Leg 1) is advanced at the early stage (Figure 9D), the swing leg speed slowly increases due to learning. As a result, the stance legs on the slow side (Legs 2 and 4) are delayed relative to the fast side at the late stage, which reduces the pitching moment to tilt the body forward and induces simultaneous foot contact between the fore and hind legs on the fast side (Legs 1 and 3), as shown in Figure 9E. These slow changes in the foot contact timings change the relative phase ${\text{Δ}}_{12}$ at the late stage.

### 4.5 Contributions of Spinal Cord and Cerebellum to Locomotor Adaptation

A split-belt treadmill imposes different speeds on the two sides of the body and highlights the functional role of interlimb coordination in adaptive locomotion. In particular, the adaptive behavior in interlimb coordination can be classified into two types, namely fast and slow adaptations. That is, the locomotion control system has two different time scales. These adaptations are primarily achieved by the contributions of different layers in the neural system, namely the spinal cord and cerebellum. The spinal cord provides motor commands through the RG and PF networks (Burke et al., 2001; Rybak et al., 2006) and modulates the commands immediately responding to sensory feedback (Grillner, 1975). This immediate modulation contributes to fast adaptation, as suggested by the fact that spinal cats walking on a split-belt treadmill show rapid adaptive behavior (Forssberg et al., 1980; Frigon et al., 2013). The cerebellum receives the efference copy from the spinal cord via the ventral spinocerebellar tract and sensory information via the dorsal spinocerebellar tract (Arshavsky et al., 1983; Fedirchuk et al., 2013). Purkinje cells provide the output from the cerebellar cortex to modulate motor commands based on error information between the sensory information predicted via the efference copy and the actual sensory information. This modification contributes to slow adaptation, as suggested from the fact that mice with Purkinje cell degeneration walking on a split-belt treadmill do not exhibit slow adaptive behavior and after-effect (Darmohray et al., 2019). The reflexive response in the spinal cord secures the ability to continue walking as the environment changes, which quickly induces asymmetric interlimb coordination. The cerebellum slowly modulates the movements under the secured condition to make walking smoother and more efficient, which slowly reduces asymmetric interlimb coordination.

Animals make predictions by evaluating various parameters to enhance their movements through learning in motor control. The cerebellum contributes to this prediction and learning. However, because it remains unclear what is predicted and how to use it in learning, modeling studies have attracted attention. In particular, learning models of human arm movements have been proposed to minimize jerk and torque change (Flash and Hogan, 1985; Uno et al., 1989). Although learning techniques, such as deep reinforcement learning, have been used to control legged robots (Hwangbo et al., 2019; Lee et al., 2020; Lillicrap et al., 2016), cerebellar learning models for locomotion remain largely unestablished. This is partly because locomotion is a whole-body movement through leg movement and posture controls and is governed by complicated dynamics, including foot contact and lift off, which change the physical constraints. In this study, we focused on the foot contact timing for prediction and learning in the cerebellar model. This is because phase modulation in response to the stimulation of nerves in the legs (Conway et al., 1987; Duysens, 1977; Frigon et al., 2010; Fujiki et al., 2019; Schomburg et al., 1998) and reflexive reaction in the absence of foot contact sensory information (Hiebert et al., 1994; van der Linden et al., 2007) suggest that sensory information related to foot contact timing play important roles in modulating locomotor behavior. In addition, ankle stiffness is predictively modulated at foot contact in split-belt treadmill walking (Ogawa et al., 2014). Moreover, climbing fiber responses of Purkinje cells, which provide error information for motor control, increase around foot contact (Yanagihara and Udo, 1994). However, the prediction and learning of foot contact timing do not necessarily lead to the adaptations observed during split-belt treadmill walking of animals. Our previous works (Fujiki et al., 2013, 2015) showed that a biped robot with our control system exhibits the fast and slow adaptations observed in humans. Furthermore, this study showed that a quadruped robot with our control system exhibits fast and slow adaptations similar to those of quadrupeds. Our results clarify the importance of foot contact timing modification through sensorimotor integration for adaptive locomotion in animals.

### 4.6 Limitations of Our Study and Future Work

In this study, we used a robotic platform to investigate the gait adaptation mechanism during quadrupedal locomotion on a split-belt treadmill. The robot mechanical system is much simpler than an animal musculoskeletal system. Furthermore, the robot body is rigid and the joints are strictly controlled by motors, whereas an animal body and joints are flexible due to control by muscles. In addition, we used a much simpler locomotion control system than the neural system used by animals. These simplifications in the robot mechanical and locomotion control systems facilitated the capture of the essential aspects of adaptive locomotion. However, they caused quantitative differences in locomotor behavior. In particular, these simplifications forced our robot to use a pace pattern, unlike the walk and trot patterns of general quadrupeds. For intact cats walking on a split-belt treadmill using a walk pattern, when the left and right belt speeds are changed, the relative phases are altered on both the contralateral and ipsilateral sides to induce asymmetric interlimb coordination, where the contralateral sides for the fore and hind legs change most significantly (D’Angelo et al., 2014). These results are not necessarily the same as our results, where the contralateral side for the fore legs showed significant changes whereas that for the hind legs showed no significant changes (Figure 4). Although some quadrupeds such as giraffes and camels use a pace pattern (Muybridge, 1957), there are no experimental data regarding how their interlimb coordination changes when they walk on a split-belt treadmill, which prevents us from verifying our results from a biological viewpoint and requires further biological studies. To overcome these limitations, musculoskeletal models, which can use similar walk and trot patterns to those used by general quadrupeds, would be useful (Fujiki et al., 2018; Toeda et al., 2020) in future studies.

Although this study focused on split-belt treadmill walking to investigate the contribution of interlimb coordination to adaptive quadrupedal locomotion, interlimb coordination plays an important role in numerous other locomotor tasks. For example, the gait transition between walk, trot, and gallop changes the phase relationship between the movements of four legs while creating and breaking the synchronization between the leg movements (Aoi et al., 2013a; Aoi et al., 2011; Fukui et al., 2019; Fukuoka et al., 2015; Masuda et al., 2021; Owaki et al., 2013; Owaki and Ishiguro, 2017). When crossing an obstacle during walking, the leading limb, which steps over the obstacle first, and the trailing limb, which steps over the obstacle after the leading limb, have different distances from the obstacle and these leg movements differ (Aoi et al., 2013b; Aoki et al., 2013). During walking along a curved path, the inner and outer limbs show different speeds (Gruntman et al., 2007). We would like to investigate the contributions of interlimb coordination to these locomotor tasks using our legged robots and mathematical models in the future.

## Data Availability

The raw data supporting the conclusions of this article will be made available by the authors, without undue reservation.

## Supplementary Material

Supplementary Movie Quadruped robot walking on a split-belt treadmill from the tied configuration to the split-belt configuration in the 5x condition.
